# Elevated Plasma Level of 8-Hydroxy-2′-deoxyguanosine Is Associated with Primary Open-Angle Glaucoma

**DOI:** 10.1155/2020/6571413

**Published:** 2020-04-25

**Authors:** Altaf A. Kondkar, Taif A. Azad, Tahira Sultan, Essam A. Osman, Faisal A. Almobarak, Saleh A. Al-Obeidan

**Affiliations:** ^1^Department of Ophthalmology, College of Medicine, King Saud University, Riyadh, Saudi Arabia; ^2^Glaucoma Research Chair in Ophthalmology, College of Medicine, King Saud University, Riyadh, Saudi Arabia

## Abstract

**Purpose:**

To determine the association between plasma 8-hydroxy-2′-deoxyguanosine (8-OHdG) levels, a marker for oxidative DNA damage, and patients with primary open-angle glaucoma (POAG) or its clinical phenotypes. Furthermore, we also examined the utility of plasma 8-OHdG as a potential biomarker in POAG.

**Materials and Methods:**

In a retrospective case-control study, plasma samples were obtained from 50 POAG cases and 45 glaucoma-free controls matched for age, sex, and ethnicity. 8-OHdG levels were measured in duplicate using an enzyme-linked immunosorbent assay (ELISA) on an automated ELISA analyzer.

**Results:**

There was no significant difference in age, sex, and systemic disease distribution between POAG cases and controls. Both mean and median 8-OHdG levels were significantly elevated in POAG cases and male subjects. The area under the receiver operating characteristic (ROC) curve value for plasma 8-OHdG was 0.653 (95% confidence interval = 0.54–0.76, *p* = 0.010). The cutoff values based on quartile distribution and ROC curve analysis showed that elevated plasma 8-OHdG significantly increased the risk of POAG by more than 4-folds. Plasma 8-OHdG had a sensitivity of 78% and specificity of 53%. In logistic regression analysis, 8-OHdG showed a significant effect on POAG outcome (*p* = 0.016) independent of age, sex, smoking, and systemic diseases. However, no significant correlation was observed between 8-OHdG and specific clinical markers of glaucoma such as intraocular pressure (*p* = 0.699), cup/disc ratio (*p* = 0.213), and the number of antiglaucoma medications (*p* = 0.603).

**Conclusion:**

The study shows that there is a significant association between elevated plasma 8-OHdG and POAG, supporting the role of systemic oxidative stress-induced DNA damage in POAG pathogenesis. However, with a high rate of false-positivity, plasma 8-OHdG may lack the ability to serve as a potential biomarker in POAG. Further studies in a much larger cohort are needed to confirm these findings.

## 1. Introduction

Glaucoma is a multifactorial and complex neurodegenerative disease that is caused by gradual apoptosis of retinal ganglion cells (RGCs) and the optic nerve head leading to irreversible blindness [1]. One of the pathologic mechanisms that may trigger apoptosis is oxidative stress via mitochondrial or endothelial damage, inflammation, and hypoxia [2]. Oxidative stress is believed to be majorly responsible for inducing molecular damage in the anterior chamber of the eye that may ultimately result in increased intraocular pressure (IOP) and subsequent manifestation of glaucoma [2].

The oxidative stress is generally induced by excessive generation of reactive oxygen species (ROS), mitochondrial dysfunction, impaired antioxidative defense mechanism, or a combination of these systems [2]. Under normal physiological conditions, there exists a balance between ROS production and clearance. However, excessive production of ROS in the cells may induce oxidative damage in the DNA, RNA, mitochondria, and other biomolecules, resulting in impairment of their cellular function(s) or cell death [2, 3]. Cell-death induced by increased oxidative stress and ROS is involved in the pathogenesis of several neurodegenerative disorders such as Alzheimer, Parkinson, prion disease, and glaucoma [4, 5]. Primary open-angle glaucoma (POAG) is an age-related disorder in which the trabecular meshwork (TM) malfunction plays a critical role [1]. In vivo studies in humans have demonstrated significantly more pronounced oxidative DNA damage in the TM cells of patients with glaucoma [6]. Furthermore, high IOP and visual field damage were both substantially proportional to the amount of oxidative DNA damage in the TM cells [7]. Besides, we have previously shown that plasma levels of total antioxidant status (TAS) were significantly lower in POAG patients as compared to nonglaucoma controls, thus supporting the role of oxidative stress-based mechanism in the pathogenesis of POAG [8]. Oxidative stress via ROS generation can induce breaks or base modifications in the DNA leading to the formation of DNA oxidation products such as 8-hydroxy-2′-deoxyguanosine (8-OHdG) [9]. 8-OHdG is considered to be a reliable marker of oxidative DNA damage that can be easily quantified.

Based on the role of oxidative stress in the pathogenesis of glaucoma and our recent findings of elevated plasma 8-OHdG levels in patients with pseudoexfoliation glaucoma (PXG) [10], we performed a case-control study to investigate the role of systemic oxidative stress-induced DNA damage in POAG. We examined an association between plasma 8-OHdG, as a marker of oxidative DNA damage, and POAG or its related clinical phenotypes. Besides, due to lack of any blood-based biomarker to assess the disease risk, we also examined the utility of plasma 8-OHdG as a potential biomarker in POAG.

## 2. Materials and Methods

### 2.1. Study Population

The study adhered to the Declaration of Helsinki principles and was approved by the institutional review board committee at the College of Medicine, King Saud University, Riyadh, Saudi Arabia (approval number: 08–657). Participants of Saudi origin with the established clinical diagnosis of POAG (*n* = 50) and healthy controls (*n* = 45) were recruited at King Abdul-Aziz University Hospital in Riyadh, Saudi Arabia, following a written informed consent. Our team consists of 3 glaucoma consultants who carry out the measurements, review the data, and perform the diagnosis. All patients underwent standardized ophthalmic examination that included intraocular pressure (IOP) measurement by Goldmann applanation tonometry mounted at the slit lamp, anterior chamber angles examination by gonioscopy, dilated pupil examination of the lens and fundus, and visual field examination by Humphrey automated field analyzer. Patients were eligible for inclusion if they met the following clinical criteria for POAG: adult-onset of the disease; intraocular pressure (IOP) ≥21 mmHg in one or both eyes before initiation of glaucoma treatment; normal-appearing, bilaterally open anterior chamber angles by gonioscopy; optic nerve appearance characteristic of the optic discs typically observed in POAG (with localized narrowing or absence of the neuro-retinal rim, with the amount of cupping exceeding the amount of pallor of the rim, and with asymmetric cupping of the optic discs); and corresponding visual field (Humphrey Field Analyzer II, Carl Zeiss Meditec, Inc., Dublin, CA, USA; using a full threshold 24–2 program) abnormalities typical of glaucoma [11]. The exclusion criteria included historical, clinical, or biochemical evidence of another possible optic neuropathic process affecting either eye, significant visual loss in both eyes not associated with glaucoma, evidence of secondary glaucoma, e.g., pigmentary dispersion syndrome, pseudoexfoliation, history of ocular trauma, steroid usage or any antioxidant supplements and vitamins, any infectious or immunomodulating diseases (rheumatoid arthritis, lupus, Crohn's disease), or refusal to participate.

Among antiglaucoma medications, beta-blockers (e.g., timolol), prostaglandin analogue (e.g., latanoprost), and carbonic anhydrase inhibitors (e.g., acetazolamide) were the most common drugs prescribed to the glaucoma patients.

The ethnically matched control group included subjects with age >20 years at recruitment; normal IOP (<21 mmHg); normal optic disk with open anterior chamber angles on examination; and no history of ocular disease(s) or eye surgeries. Other information related to the history of systemic diseases, family history, and smoking status was obtained from medical records or personal interviews. Subjects taking any antioxidant supplements, vitamins, or having any infectious or immunomodulating diseases (rheumatoid arthritis, lupus, and Crohn's disease), or refusing to participate were excluded.

### 2.2. Levels of Plasma 8-Hydroxy 2′-deoxyguanosine

Estimation of 8-OHdG levels from plasma was performed using a commercial competitive sandwich enzyme-linked immunosorbent assay (ELISA) kit (Trevigen, Gaithersburg, MD, USA). The assay was performed in duplicate as per the manufacturer's instructions on an automated ELISA analyzer, ChemWell-T (Awareness Technology Inc., FL, USA). The concentrations of 8-OHdG levels were determined from a standard curve and expressed in ng/mL.

### 2.3. Statistical Analysis

Statistical analysis was performed with SPSS version 19.0 (IBM Corp., Armonk, New York, USA) and Stat View software version 5.0 (SAS Institute, Cary, NC, USA). Data were tabulated as mean, median, frequency, and percentages. Normality testing for 8-OHdG levels was done using Kolmogorov–Smirnov test. Mean differences between groups were tested by independent sample *t*-test. Mann–Whitney *U* test was used to compare median values between the patients and controls. The categorical variables were tested by Chi-square test. The associated risk was expressed as odds ratio (OR) and 95% confidence interval (CI). The correlation testing was done using Spearman's method. A binary logistic regression analysis was performed to estimate the impact and effect of mean 8-OHdG level and other risk factors on disease outcome. To determine clinical utility of 8-OHdG, area under the receiver operating characteristic (ROC) curve (AUC), sensitivity (true-positive), and specificity (true-negative) of the assay were examined by ROC curve and analyzed by Mann–Whitney test. All statistical tests were two-sided, and *p* value <0.05 was considered statistically significant.

## 3. Results

### 3.1. Study Population and Plasma 8-OHdG Levels

**Figure 1:**
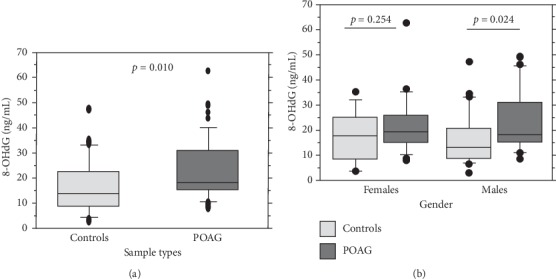
Box plot showing the distribution of 8-hydroxy-2′-deoxyguanosine levels: (a) between primary open-angle glaucoma cases and controls and (b) according to gender in cases and controls. 8-OHdG, 8-hydroxy-2′-deoxyguanosine; POAG, primary open-angle glaucoma.

As shown in Table 1, the study groups showed no significant difference in terms of age, gender, smoking habits, and other systemic disease conditions (*p* > 0.05). The prevalence of family history of glaucoma was significantly more in the patient group (*p*=0.012). Besides, the mean 8-OHdG level was significantly elevated in the POAG patients (22.61 ± 12.05 ng/mL) than the controls (16.95 ± 10.66 ng/mL; *p*=0.018). Also, the median 8-OHdG levels were significantly high (*p*=0.010) between controls (13.78 ng/mL) and POAG (18.53 pg/mL). Similarly, gender-stratification showed that both the mean and median levels of 8-OHdG were significantly elevated in POAG males but not in females (Table 1).  Figure 1 shows the box-plot distribution of plasma 8-OHdG based on sample types and gender in POAG cases and controls. The clinical characteristics of POAG patients are shown in Table 2, and a representative imaging data of a POAG patient is shown in Supplementary Figure 1.

### 3.2. Plasma 8-OHdG and Risk of POAG

To evaluate the risk of POAG at different levels of 8-OHdG (dose-related trend), the uncategorized data (not as cases and controls) were dichotomized into quartiles to identify two cutoff values of 11.18 ng/mL (first quartile or the 25^th^ percentile) and 26.60 (third quartile or the 75^th^ percentile). Subjects were then categorized into three groups by using these two cutoff values as shown in Table 3. Quartile distribution data showed that there was a significant additive effect of increasing levels of 8-OHdG on POAG outcome (*χ*^2^ = 8.58, df = 2; *p*=0.014). Besides, compared to 8-OHdG, levels less than the first quartile or the 25^th^ percentile (<11.18 ng/mL), subjects with interquartile (25^th^–75^th^ percentile) levels, and those higher than the third quartile (75^th^ percentile) exhibited a significantly increased risk of POAG. Similarly, the ROC curve also allows identification of best cutoff values to maximize sensitivity and specificity [12]. Based on ROC curve analysis, a cutoff value of 14.8 ng/mL also exhibited 4-fold increased risk of POAG (*p*=0.002) (Table 2).

### 3.3. 8-OHdG as a Biomarker

**Figure 2:**
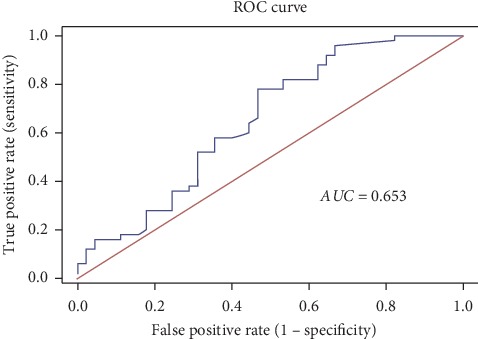
ROC curve analysis. AUC, area under the receiver operator characteristic curve; ROC, receiver operator characteristic.

ROC curve was generated to evaluate the potential of plasma 8-OHdG to discriminate between POAG cases and controls (Figure 2). The analysis yielded a significant (*p*=0.010) AUC value of 0.653 (95% CI = 0.541–0.765). The accuracy of a test marker is directly proportional to the AUC value which can vary between 0.5 and 1.0, with 0.5 indicating poor accuracy, 0.5–0.7, moderate, 0.7–0.9, highly accurate, and 1.0 suggesting a best-fit, thus indicating that plasma 8-OHdG was a significant moderate discriminator between POAG cases and controls. The sensitivity, specificity, and other characteristics of 8-OHdG as a biomarker based on the ROC curve threshold are shown in Table 4. At a cutoff value of 14.8 ng/mL, plasma 8-OHdG had a sensitivity of 78% and specificity of 53%.

### 3.4. Plasma 8-OHdG and Other Potential Confounders

A binary logistic regression analysis was performed to investigate the effect of multiple factors such as age, sex, systemic diseases, smoking, and plasma 8-OHdG levels in patients with POAG, using diseased/nondiseased as a dependent variable (outcome). Except for plasma 8-OHdG (*p*=0.016), none of the other confounding variables showed any significant effect on the disease outcome (Table 5).

### 3.5. Correlation between 8-OHdG and Glaucoma Indices in POAG Patients

There was no significant correlation between 8-OHdG and age, sex, and other clinical phenotypic markers of disease such as IOP, cup/disc ratio, and the number of antiglaucoma medication (Table 6).

## 4. Discussion

Both endogenous (normal cellular metabolism) and exogenous factors (e.g., UV) can generate ROS, which as a result of impaired pro-oxidant and antioxidant balance, can cause DNA damage and contribute significantly to glaucomatous neurodegeneration [13]. Base guanine is considered to be highly susceptible to oxidative modifications due to its low redox potential, and its most common byproduct 8-OHdG is believed to be an excellent marker for oxidative stress-induced DNA damage [14]. Oxidative DNA damage constitutes a significant threat to genetic integrity and thus has been implicated in the pathogenesis of complex human diseases such as cancer and neurodegenerative disorders, including glaucoma [2, 4, 15, 16]. In this study, we report an association between increased levels of systemic 8-OHdG, a marker of oxidative stress-induced DNA damage, and POAG.

Several studies in the past have provided a vital link between increased levels of 8-OHdG and glaucomatous optic neuropathy [6, 7, 17]. Early studies by Izzotti et al. demonstrated a more than 3-fold increase in 8-OHdG levels in the TM cells of glaucoma patients with the amount of oxidative DNA damage correlating to IOP increase and visual filed loss, thereby providing convincing evidence for the role of oxidative DNA damage in glaucoma [6, 7]. Since then, studies in human samples have also consistently reported an increase in 8-OHdG levels in different forms of glaucoma. In a small study consisting of POAG and PXG, both aqueous humor (AH) and serum samples were elevated in cases compared to controls [17]. Similarly, Mohanty and colleagues reported that both plasma and AH 8-OHdG were elevated in POAG patients as compared to cataract controls [18]. Moreover, the increase was attributed to reduced expression of DNA repair enzymes of the base excision repair pathway [18], and a strong positive correlation between plasma 8-OHdG levels and AH 8-OHdG levels were reported, suggesting that systemic 8-OHdG levels could be predictive of local 8-OHdG levels in the eye [18]. In another recent study by Mumcu et al. increased 8-OHdG levels, as measured by high-performance liquid chromatography, and decreased paraoxonase-1 activity were associated with POAG [19]. Likewise, studies by Yuki et al. have reported increased urinary 8-OHdG levels to be associated with glaucomatous visual field progression in subjects with normal tension glaucoma [20, 21], and Chang and colleagues have reported elevated serum 8-OHdG in patients with primary angle-closure glaucoma of Chinese origin [22]. Besides, 8-OHdG levels have also been reported as potential diagnostic marker in other neurodegenerative diseases such as dementia (e.g., Alzheimer's disease) and Parkinson's disease (PD). In an excellent study by Choromanska and colleagues, significantly increased oxidative damage (measured as 8-OHdG among others) and decreased antioxidant status were reported in stimulated and nonstimulated saliva. The results of the study suggested that changes in salivary redox homeostasis are independent of systemic (plasma/erythrocytes) changes in the course of dementia [23]. Likewise, cerebrospinal fluid 8-OHdG levels were significantly increased in nondemented PD patients suggesting that 8-OHdG levels could potentially complement neurochemically supported diagnosis of PD [24].

In concordance with these reports, our findings show that both the mean and median levels of 8-OHdG are significantly increased in our POAG cohort and increase the risk of POAG by more than 4-folds. However, plasma 8-OHdG showed no significant correlation with IOP, cup/disc ratio, and number of antiglaucoma medication, indicating a lack of association with these clinical phenotypes or markers used to assess disease severity. Furthermore, the ROC curve analysis showed that plasma 8-OHdG, as a biomarker, has good sensitivity but low specificity. This indicates that plasma 8-OHdG may be able to predict disease (POAG) with a high rate of false-positivity and thus may not serve as a potential circulating biomarker in POAG. It is noteworthy that since 8-OHdG is an oxidative stress biomarker, its levels can be influenced by aging, infectious diseases, smoking habits, and other systemic diseases such as diabetes, hypertension, or coronary disease [3, 25–27]. Thus, care was taken to exclude individuals with infectious or autoimmune diseases or those taking dietary vitamins and supplements. Besides, the control group showed no significant deviation than the patient group in terms of age, ethnicity, gender, smoking habits, and systemic disease status. Furthermore, logistic regression analysis indicated that the significant effect of plasma 8-OHdG on POAG risk was independent of age, gender, smoking, or systemic diseases, thereby suggesting that elevated plasma 8-OHdG could be more plausibly related to POAG condition rather than to any of these confounding variables. Clearly, further investigations are needed in a much larger cohort to confirm these findings. However, the observed increase in the plasma levels of 8-OHdG clearly suggests an increased oxidative stress-induced DNA damage and its plausible association with glaucomatous degeneration and is in agreement with our previous observation in PXG patients of Saudi origin [10].

The role of 8-OHdG in glaucoma development and progression is still speculative. Local increase of oxidative DNA damage in the eye (as shown in TM cells) [6] can cause TM degeneration, hypoxia, and rise in IOP. These can lead to the clinical onset of glaucoma [16]. Besides, failure to repair DNA lesions can result in transversion mutations which can have serious biological consequences [28]. Oxidative DNA damage may accelerate telomere shortening and cause cell senescence associated with aging and degenerative diseases [28, 29]; or oxidative stress through the formation of 8-OHdG may induce epigenetic instability which can activate oncogenes or inactive tumor suppressor genes [15, 28]. The pathophysiological effects of oxidative stress and ROS in glaucoma through glial cell damage, autophagy, nuclear-kappa B activation (signaling), nitrite stress, and alterations in ocular hemodynamics to propagate inflammation and optic nerve damage or RGC death are also well documented (as reviewed elsewhere) [2]. Oxidative stress has also been strongly linked to mitochondrial abnormalities and reduced total antioxidant status as has been consistently proven in POAG [8, 30] and PXG [31, 32], thereby substantiating our current finding that oxidative stress-induced DNA damage may have a significant role to play in the pathogenesis of POAG and that the high levels of 8-OHdG observed in our study may plausibly contribute to POAG pathogenesis by similar mechanism(s). Paradoxically, exogenous (synthetic) 8-OHdG has been suggested as a potential candidate for the treatment and prevention of inflammation-based gastrointestinal diseases and cancer [33].

The study has certain limitations. The systemic increase in 8-OHdG may not reflect the actual microenvironment of the cells/tissues in the anterior chamber of the eye which are continuously exposed to ROS and are more related to the disease. To achieve this, analysis of AH samples would be appropriate. Besides, the study is purely descriptive with no mechanistic evidence to suggest causal implications of high 8-OHdG in POAG. Lastly, the study is limited in its capacity to evaluate 8-OHdG association with disease severity or advancement due to its relatively small sample size for subgroup analyses and low number of patient samples in the lowest-quartile of 8-OHdG. This may also explain the lack of correlation of plasma 8-OHdG with IOP and cup/disc ratio; or its lack of association in females (subgroup). An investigation in a large population-based cohort would certainly confirm these findings. However, based on the data observed in this study, an actual mean difference of 6 ng/mL in the POAG and controls with a standard deviation of 10, the probability (power) to detect an association with POAG is >0.8. The type I error probability associated with this test for the null hypothesis is 0.05.

## 5. Conclusion

In conclusion, the study provides an evidence for a significant association between plasma 8-OHdG and POAG and suggests that elevated levels of systemic 8-OHdG may be a significant risk factor for POAG. The findings add to the growing body of evidence supporting the role of systemic oxidative stress-induced DNA damage in the development or progression of POAG. However, plasma 8-OHdG did not show any significant correlation with clinical markers of POAG (e.g., IOP and cup/disc ratio) but showed moderate ability to discriminate between cases and controls, and exhibited a high rate of false-positivity. Thus, plasma 8-OHdG may not serve as a potential clinical biomarker in POAG. Further investigations in a much larger cohort are needed to validate these results and assess the association/correlation between systemic oxidative DNA damage status, glaucoma severity, and extent of visual field damage in POAG.

## Figures and Tables

**Table 1:** Demographic, systemic disease status, and 8-hydroxy-2′-deoxyguanosine levels between patients with primary open-angle glaucoma and healthy controls.

Variables	Controls (*n* = 45)	POAG (*n* = 50)	*p* value
Age in years, mean (SD)	59.9 (8.3)	62.3 (9.2)	0.191^a^
Male/female, *n*	30/15	28/22	0.287^b^
Systemic diseases, *n* (%)			
Diabetes mellitus	6 (13.3)	8 (16.0)	0.714^b^
Hypertension	5 (11.1)	7 (14.0)	0.672^b^
Coronary artery disease	2 (4.4)	4 (8.0)	0.477^b^
Hypercholesterolemia	2 (4.4)	3 (6.0)	0.735^b^
Family history of glaucoma	1 (2.2)	9 (18.0)	0.012^b^
Smokers	5 (11.1)	7 (14.0)	0.672^b^
8-OHdG levels, ng/mL			
Mean (SD)	16.95 (10.66)	22.61 (12.05)	0.018^a^
Median	13.78	18.35	0.010^c^
Males			
Mean (SD)	17.02 (11.02)	23.15 (12.24)	0.049^a^
Median	13.12	18.08	0.024^c^
Females			
Mean (SD)	16.80 (10.29)	21.92 (12.06)	0.188^a^
Median	17.97	19.24	0.254^c^

Note. ^a^Independent sample *t*-test (two-tailed); ^b^Chi^2^ test; ^c^Mann–Whitney *U* test. 8-OHdG, 8-hydroxy-2′-deoxyguanosine; POAG, primary open-angle glaucoma.

**Table 2:** Clinical characteristics of primary open-angle glaucoma patients.

Characteristics	POAG
Age in years, mean (SD)	62.3 (9.2)
Male/female, *n*	28/22
^a^Intraocular pressure mmHg, mean (SD)	23.8 (8.0)
Cup/disc ratio	0.78 (0.15)
Number of antiglaucoma medications, mean (SD)	1.98 (0.90)

^a^Baseline corrected for central corneal thickness. POAG, primary open-angle glaucoma.

**Table 3:** Plasma levels of 8-hydroxy-2′-deoxyguanosine and the risk of primary open-angle glaucoma.

8-OHdG cutoff ng/mL	Controls no. (%)	POAG no. (%)	Odds ratio (95% confidence interval)	*p* value^a^
By quartiles^†*∗*^				
<11.18	17 (33.3)	6 (12.0)	Reference	–
11.18–26.60	19 (26.6)	30 (60.0)	4.47 (1.49 – 13.35)	0.005
>26.60	9 (40.0)	14 (28.0)	4.40 (1.26 – 15.41)	0.017

By ROC curve				
<14.80	24 (53.3)	11 (22.0)	Reference	
≥14.80	21 (46.6)	39 (78.0)	4.05 (1.66 – 9.86)	0.002

Note. ^a^Pearson's Chi^2^ test; ^†^first quartile (<25^th^ percentile); interquartile (25^th^–75^th^ percentile); third quartile (>75^th^ percentile). ^*∗*^Overall Chi^2^ = 8.58, df = 2, *p* < 0.014. 8-OHdG, 8-hydroxy-2′-deoxyguanosine; POAG, primary open-angle glaucoma; ROC, receiver operator characteristic.

**Table 4:** ROC curve characteristics of plasma 8-hydroxy-2′-deoxyguanosine as a biomarker in POAG.

Characteristics	Value	95% confidence interval
Cutoff value, ng/mL	≥14.8	–
AUC (SE)	0.653 (0.057)	0.54 – 0.76
*p* value	0.010	–
Sensitivity, %	78.0	64.0 – 88.4
Specificity, %	53.3	37.8 – 68.3
Positive likelihood ratio	1.67	1.18 – 2.36
Negative likelihood ratio	0.41	0.23 – 0.74
Positive predictive value, %	65.0	56.8 – 72.4
Negative predictive value, %	68.5	54.7 – 79.7
Accuracy, %	66.3	55.9 – 75.7

AUC, area under the receiving operating characteristics curve; POAG, primary open-angle glaucoma; ROC, receiver operating characteristic.

**Table 5:** Binary logistic regression analysis to assess the effect of 8-hydroxy-2′-deoxyguanosine levels and other potential confounders on disease outcome.

Variables	B	SE	Odds ratio (95% CI)	*p* value
Age	0.039	0.025	1.04 (0.99 – 1.09)	0.120
Sex^a^	–0.569	0.449	0.56 (0.23 – 1.36)	0.205
Diabetes	0.139	0.715	1.15 (0.28 – 4.66)	0.846
Hypertension	0.066	0.804	1.06 (0.22 – 5.16)	0.935
Heart disease	–0.009	1.060	0.99 (0.12 – 7.9)	0.994
Hypercholesterolemia	–0.633	1.105	0.53 (0.06 – 4.63)	0.567
Smoking	0.557	0.727	1.74 (0.42 – 7.25)	0.443
8-OHdG	0.052	0.022	1.05 (1.01 – 1.10)	0.016
Constant	–2.79	1.662	0.06	0.092

^a^Females as reference. 8-OHdG, 8-hydroxy-2′-deoxyguanosine.

**Table 6:** Correlation analysis between 8-hydroxy-2′-deoxyguanosine levels and glaucoma specific clinical indices in patients.

Variables	*R*	*p* value
Age	–0.067	0.643
Sex	0.008	0.953
Intraocular pressure, mmHg	0.056	0.699
Cup/disc ratio	–0.179	0.213
Number of antiglaucoma medications	0.075	0.603

*R*, Spearman's correlation coefficient.

## Data Availability

The data supporting the conclusions of this article are all presented within the report.

## Supplementary Materials

Supplementary Materials Supplementary Figure 1: (a) optic nerve with advanced damage and (b) corresponding advanced visual field defect with HVF, 24–2 strategy in a POAG patient.
