# Supplementary figures and images for: Elevated Plasma Level of 8-Hydroxy-2′-deoxyguanosine Is Associated with Primary Open-Angle Glaucoma
Source: J Ophthalmol. 2020 Apr 25;2020:6571413. doi: 10.1155/2020/6571413 (PMC7201519; doi:10.1155/2020/6571413)

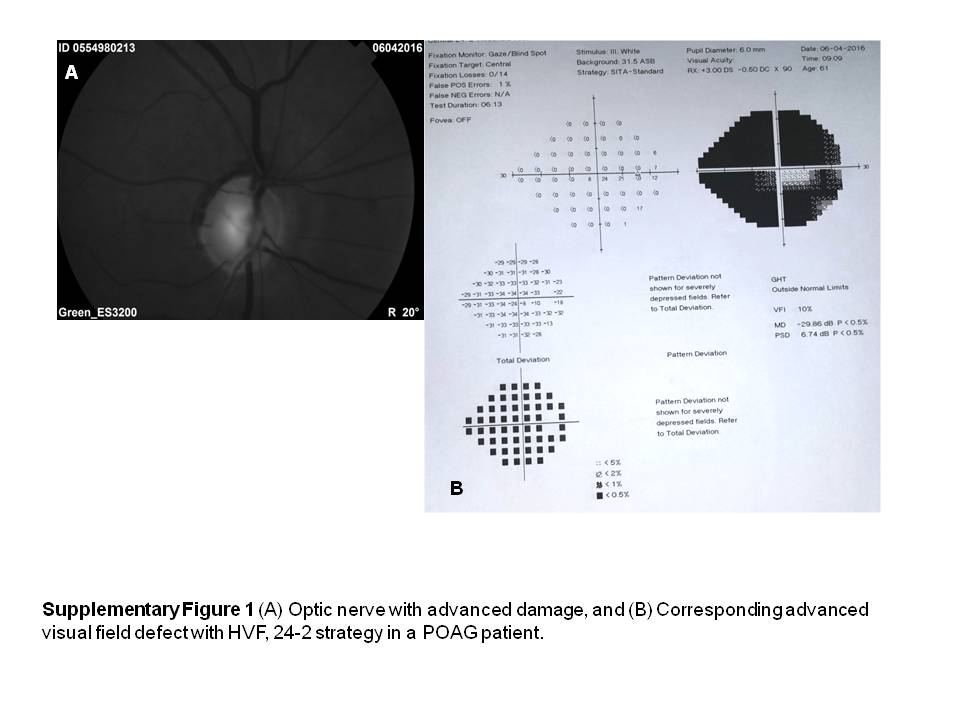

Supplement: Supplementary Materials — Supplementary Figure 1: (a) optic nerve with advanced damage and (b) corresponding advanced visual field defect with HVF, 24–2 strategy in a POAG patient. [file 6571413.f1.jpg]
